# Glabridin content and metabolic effects of liquorice confectionaries: A secondary analysis of a randomized crossover trial in young adults

**DOI:** 10.1371/journal.pone.0358710

**Published:** 2026-09-22

**Authors:** Anna Åstrand, Annelie Joelsson, Karin Rådholm, Fredrik H. Nyström, Peder af Geijerstam

**Affiliations:** 1 Division of Clinical Chemistry and Pharmacology, Department of Biomedical and Clinical Sciences, Linköping University, Linköping, Sweden; 2 Primary Healthcare Center Cityhälsan Centrum, Region Östergötland, Norrköping, Sweden; 3 Department of Health, Medicine and Caring Sciences, Linköping University, Linköping, Sweden; 4 Primary Healthcare Center Kärna, Region Östergötland, Linköping, Sweden; 5 The George Institute for Global Health, University of New South Wales, Sydney, Australia; Sungkyunkwan University - Suwon Campus: Sungkyunkwan University - Natural Sciences Campus, KOREA, REPUBLIC OF

## Abstract

**Background:**

Liquorice, a popular confectionary product, contains several bioactive compounds. Amongst them, glycyrrhizic acid is associated with increased blood pressure, and glabridin is associated with anti-inflammatory and glucose-lowering effects. The aim was to assess the association between short term liquorice intake at a dose known to elevate blood pressure, and markers of dysglycemia, blood lipids, high-sensitivity C-reactive protein (hsCRP), and calcium metabolism.

**Methods:**

In a 2x2 crossover study, 28 healthy adults were randomized to consume liquorice or a control product in alternating 2-week periods, with measurements of blood glycated haemoglobin A_1c_ as well as plasma glucose, calcium ion, parathyroid hormone, and lipids at the end of each period. Liquorice glabridin content was determined via LC-MSMS analysis.

**Results:**

The liquorice contained 0.25 ± 0.14 µg glabridin per mg. The plasma hsCRP value increased by 32.5% (95% CI −9.7 to 74.6) during the intervention period vs 311.5% (95% CI −40.8 to 663.7) during the control period, *P* = .045. No effects on the other markers were observed.

**Conclusion:**

Liquorice intake did not improve metabolic markers but attenuated hsCRP elevation. The potential metabolic benefits of glabridin are unlikely to outweigh the hypertensive effects when consumed as whole liquorice.

## Introduction

Liquorice, produced from the root of the *Glycyrrhiza* species, is globally used as a flavouring, phytomedicine and confectionaries [1,2]. The main bioactive component of liquorice, glycyrrhizic acid, is known to raise blood pressure (BP) in humans. Previously we found that even a daily intake of 100 mg glycyrrhizic acid, found in 3.3 grams of whole liquorice, raised systolic BP by 3.1 mmHg [3]. However, liquorice contains several other active substances. An example is the antioxidant isoflavonoid glabridin, first characterized in 1976 and unique to *Glycyrrhiza glabra* [4,5].

Glabridin has been suggested to possess health-beneficial properties. Interestingly, there are studies showing that glabridin has potential cardiovascular protective effects, including inhibition of low-density lipoprotein (LDL) oxidation and anti-inflammatory properties [6–8]. In mice, glabridin has also shown a comparable glucose-lowering effect to that of common antidiabetic medications [9]. In vitro, glabridin has been associated with antitumor effects [10–13], increased estrogenic activity [14], as well as reduced platelet aggregation [15], oxidative stress [16,17], and myofibroblast activity [18]. Glabridin may also affect bone metabolism, as liquorice has been shown to increase levels of parathyroid hormone (PTH) [19]. Despite these suggested effects, few *in vivo* studies have examined the effects of glabridin, and these have primarily reported reductions in cholesterol levels and LDL oxidation, S1 Table [20–22]. Furthermore, no randomized controlled trial has examined the effect of liquorice on these markers at the low dose evaluated in our study [3].

Liquorice confectionaries are a dietary source of glabridin, but whether the hypertensive effects of whole liquorice intake can be outweighed by potential beneficial effects on metabolism, blood lipids, and systemic inflammation remains unknown. The aim of this study was therefore to assess the association between a daily intake of 3.3 g of liquorice and markers of dysglycemia, blood lipids, systemic inflammation, and calcium metabolism. Also, given the reported link between glabridin and these markers, we aimed to quantify the glabridin content in the administered liquorice using liquid-chromatography tandem mass spectrometry (LC-MSMS).

## Methods and materials

### The metabolic effects of glabridin in young adults

This study was a secondary analysis of a crossover study. The methodology has been previously described in detail [3]. In brief, 28 volunteers aged 18–30 years were recruited from 3 January until 11 April 2023 in Östergötland County, Sweden. Exclusion criteria included known kidney, liver, or endocrine disease, as well as treatment with hormones (including oral contraceptives but not including intrauterine devices). The study had a non-blinded, 2-treatment, 2-period, 2-sequence (2x2) crossover design. After a run-in period, participants were randomized using drawing ballots to alternate between liquorice and a control product in 2-week periods, separated by 2-week wash-out periods.

### Intervention and control products

Liquorice pastilles (from Nature Med S.r.l., Cosenza, Italy), produced from *Glycyrrhiza glabra* only and with a manufacturer specified content of 4% sugars was used as intervention product. Sugar-free salty liquorice flavoured confectionaries (manufactured by Troll-Gott Konfektyr AB, Årjäng, Sweden) without any declared content of raw liquorice were used as control [23,24]. The energy content of the products was similar (S2 Table), and the products have previously been described in detail [1]. During the intervention period, each participant was instructed to consume 3.3 g of liquorice pastilles daily. During the control period, each participant was asked to consume 2.9 g of the control product daily.

### Anthropometric measurements and questionnaires

All visits took place at a study centre in Norrköping, Östergötland County, Sweden, during 2023. Participants were asked to not use any liquorice for at least 4 weeks before inclusion. Baseline measurements included height, weight, and a questionnaire assessing age, sex, tobacco and alcohol use, and physical activity habits. At the end of each study period, weight measurements were repeated.

Blood samples were drawn at baseline and at the end of the control and intervention periods, between 8 and 9 am after an over-night fast, with participants in the seated position after at least 5 minutes of rest. Lipid profile and high-sensitivity C-reactive protein (hsCRP) were analysed from 3 mL plasma tubes with lithium heparin and gel. Calcium ion and insulin were analysed from plasma tubes with serum gel and clot activator of 4 and 3.5 mL respectively. Glycated haemoglobin A_1c_ (HbA_1c_) was analysed from 3 mL blood tubes with K_2_ EDTA; and glucose was analysed from 3 mL FC Mixture tubes containing citrate, natrium fluoride and Na_2_EDTA.

Plasma glucose was measured using a hexokinase-based enzymatic photometric method on Cobas c303/c503 (Roche Diagnostics, Mannheim, Germany). Serum insulin was determined by electrochemiluminescence immunoassay (ECLIA) on Cobas e801 (Roche Diagnostics, Mannheim, Germany). Plasma lipids were analysed using an enzymatic colorimetric photometric method on Cobas c503 (Roche Diagnostics, Mannheim, Germany) or a Trinder-reaction photometric method on Indiko (Thermo Fisher Scientific, Waltham, MA, USA). High-sensitivity CRP in plasma was analysed by immunoturbidimetric photometry on Cobas c503 (Roche Diagnostics, Mannheim, Germany). Calcium ion in serum was measured using ion-selective electrode potentiometry on ABL 825 Flex, ABL 90, or ABL 800 (Radiometer, Copenhagen, Denmark). Parathyroid hormone in plasma was quantified using ECLIA on Cobas e801 (Roche Diagnostics, Mannheim, Germany). All blood sample were analysed by validated methods at the ISO/IEC 17025-accredited laboratory Diagnostikcentrum i Östergötland, Linköping, Sweden.

### Statistical analyses

Normality of distributions was assessed using the Shapiro-Wilk test. Continuous variables were shown as the mean and standard deviation, and differences between groups were tested using a 2-sided Wilcoxon rank-sum test. Categorical variables were shown as the frequency and percentage, and differences between groups were tested using a Chi-squared test.

Results were evaluated by comparing the mean differences between the run-in period and the intervention and control periods respectively and tested using a 2-sided paired *t* test for variables with a normal distribution and a 2-sided paired Wilcoxon signed-rank test for variables with a skewed distribution. Individual hsCRP values were further visualized in a sex-stratified paired dot-and-line plot to facilitate interpretation.

Data were collected using the REDCap 13.1.35 digital platform (Vanderbilt University, Nashville, TN, USA) and analyses were made using R version 4.3.3 (R Core Team, Vienna, Austria) and R Studio version 2023.12.1 + 402 (Posit Software, Boston, MA, USA). All statistical tests were 2-tailed, with *P* < .05 considered statistically significant. This study was a secondary analysis of a previously published dataset [3], and the sample size was therefore not derived from a power calculation for the outcomes of this study.

### Quantification of glabridin in liquorice confectionaries

#### Chemicals and reagents.

A certified reference standard (≥98% pure) of Glabridin was purchased from Sigma-Aldrich (St Louis, MO, USA). LC–MS grade acetonitrile and methanol were obtained from Merck (Darmstadt, Germany). Formic acid was procured from Honeywell Fluka (Brussels, Belgium) and 99.5% ethanol was purchased from Solveco (Rosersberg, Sweden). Ultra-pure water was produced in-house using a Milli-Q Q-POD unit from Merck Millipore (Billerica, MA, USA).

#### Liquid chromatography tandem mass spectrometry analysis.

For LC-MSMS analysis a Waters TQ-Absolute instrument (Waters, Solna, Sweden) was used. Mobile phases were 0.01% formic acid in MQ water (A) and 0.01% formic acid in acetonitrile (B). As stationary phase a Waters ACQUITY UPLC BEH C18 Column (50 mm x 2.1 mm, 1.7 μm) was used in combination with a Waters ACQUITY UPLC BEH C18 VanGuard Pre-column (5 mm x 2.1 mm, 1.7 μm). The mobile-phase gradient used was as follows: 40% mobile-phase B was ramped up to 60% within 4 minutes, a 1-minute wash at 90% mobile-phase B and 2-minutes equilibration at 40% B follows. The flowrate was set to 500 μL/min. The column temperature was maintained at 40°C, and the injection volume was set to 1 μL. The samples were extracted and analysed together with a freshly prepared standard curve each day of analysis. Mass detection was performed by a Waters Xevo TQ Absolute (Waters, Stockholm, Sweden) mass spectrometer using electrospray ionization (ESI) interface in negative (ES-) mode. Multiple reaction monitoring method parameters were as follows: dwell time 25 ms, cone voltage 30 V, collision energy 20 eV. The target transition, m/z 323.1 → 201.3, was monitored for 8 minutes.

Validation was performed according to the validation scheme by Peters et al. for new analytical methods to be used for analysis of rare analytes [25]. The calibration function was tested on 5 levels with duplicate standards at: 1, 2, 5, 10, 20 ng/mL and found to be linear (y=0.0012x+0.143) with an R^2^ = 0.9988. The repeatability was tested on 2 levels (1 and 10 ng/mL) with 5 repeats each and found to be 3.78% at 1 ng/mL and 3.8% 10 ng/mL respectively. The accuracy was 103% ± 3% at 1 ng/mL and 104% ± 4% at 10 ng/mL. Signal to noise at 1 ng/mL was determined to be 36 and is considered the limit of quantification (LOQ) of the method.

#### Sample preparation and data analysis.

One sample of each of the confectionaries were randomly selected for analysis. The pastille (liquorice) was grinded whereafter 10 mg, in triplicates, were weighted and transferred to glass vials for extraction. The control product was cut in small pieces from the middle of the candy and 10 mg in triplicates were weighted and transferred to glass vials. To avoid cross-contamination, the 2 types of confectionaries were handled separately. All samples were extracted in 500 µL 30:70% ethanol: Milli-Q Water for 1 hour at 50°C thereafter they were transferred to injection vials. Prior to LC-MSMS analysis, the liquorice samples were diluted 200 times.

The described extraction method was previously published by Tian et al and reported 72.5% recovery for glabridin [26]. The results were calculated using a freshly prepared standard curve, assumed 72.5% glabridin extraction and expressed as µg glabridin/mg liquorice.

### Ethical considerations

The study complied with the declaration of Helsinki and was approved by the Swedish Ethical Review Authority (Dnr 2022-06163-01). All participants gave written, informed consent prior to participation. For this secondary analysis no extra sampling was performed. The original study was registered at ClinicalTrials.gov before initiation (registration number NCT05661721).

## Results

**Fig 1:**
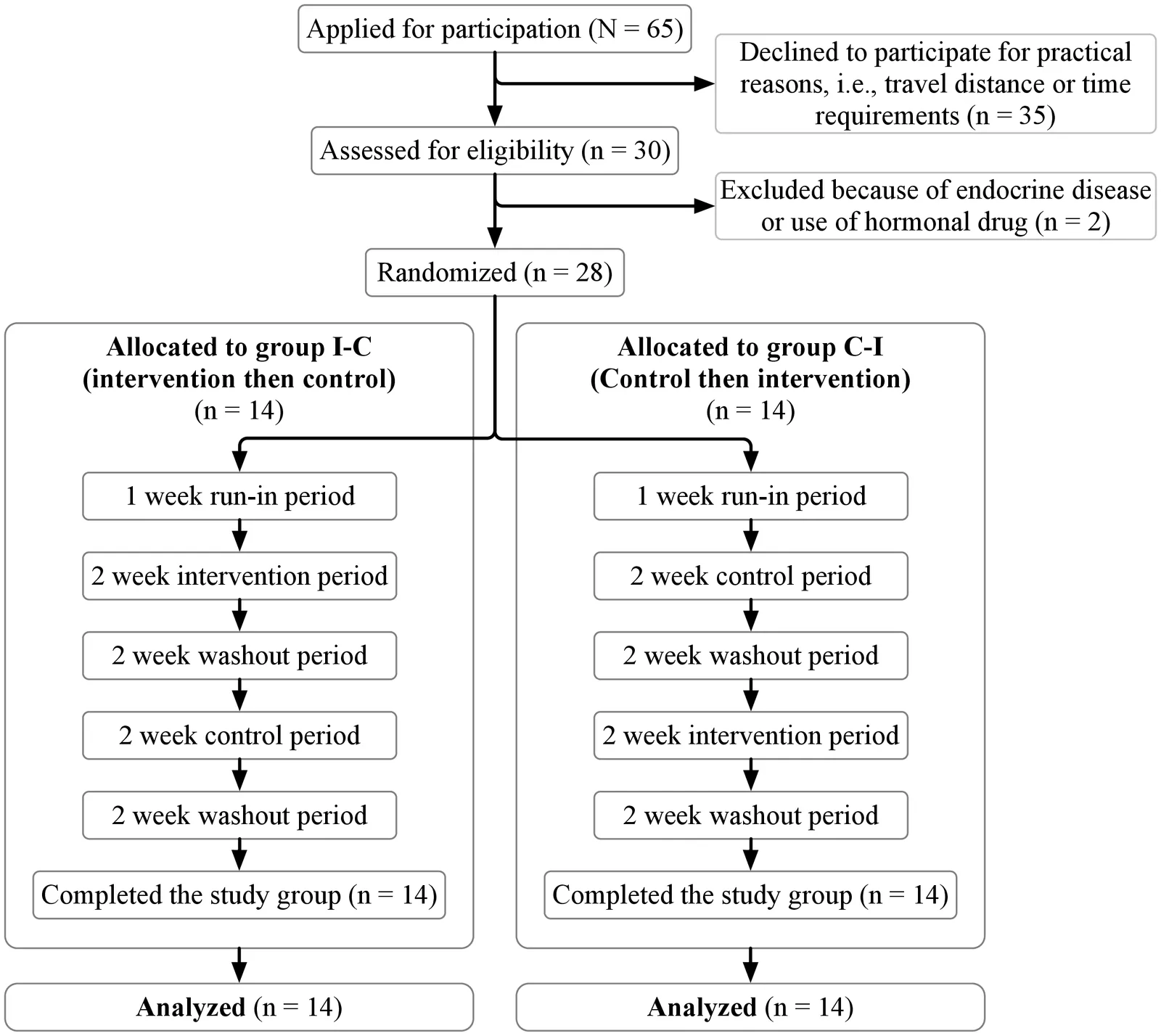
Study flow chart.

All 28 participants completed the study, Fig 1. Of participants, 14 (50%) were men. The mean age was 24.6 (standard deviation, SD 2.9) years, and the mean body mass index was 24.2 (SD 4.7) kg/m^2^. Laboratory results were similar between the 2 groups (intervention then control and control then intervention) at baseline except for plasma PTH, which was 4.3 (Q1-Q3 3.7–4.8) vs 3.2 (Q1-Q3 2.8–4.1) pmol/L, *P* = .029 (Table 1).

**Table 1:** Baseline characteristics of 28 healthy adults according to group.

	All participants, N = 28	Intervention then control, n = 14	Control then intervention, n = 14	*P*
Sex, men, n (%)	14 (50.0)	7 (50.0)	7 (50.0)	NA
Age (y), mean (SD)	24.6 (2.9)	23.7 (2.9)	25.4 (2.7)	.099
Weight (kg), mean (SD)	75.6 (15.3)	72.6 (15.1)	78.5 (15.4)	.329
Body mass index (kg/m^2^), mean (SD)	24.2 (4.7)	23.3 (3.8)	25.1 (5.5)	.454
Current snuff user, n (%)	10 (35.7)	2 (14.3)	8 (57.1)	.018
AUDIT (points), median (Q1-Q3)	5.5 (3.0-7.0)	5.0 (3.0-7.0)	5.5 (4.0-6.0)	.889
Physical activity at least 3–5 times per week, n (%)	23 (82.1)	12 (85.7)	11 (78.6)	.622
Sibling or parent with hypertension, n (%)	4 (14.3)	1 (7.1)	3 (21.4)	.280
Sibling or parent with diabetes, n (%)	2 (7.1)	1 (7.1)	1 (7.1)	>.99
Sibling or parent with myocardial infarction or stroke, n (%)	1 (3.6)	1 (7.1)	0	.309
Plasma glucose (mmol/L), median (Q1-Q3)	5.3 (5.0-5.6)	5.3 (4.9-5.6)	5.3 (5.1-5.6)	.799
Blood HbA_1c_ (mmol/mol), median (Q1-Q3)	33.0 (31.0-35.0)	33.5 (32.3-34.8)	32.0 (31.0-34.5)	.404
Serum insulin (mIU/L), median (Q1-Q3)	8.6 (5.9-11.3)	8.6 (8.1-11.8)	7.9 (5.5-9.9)	.448
Plasma cholesterol (mmol/L), median (Q1-Q3)	4.0 (3.6-4.4)	4.0 (3.6-4.3)	4.0 (3.5-4.7)	.800
Plasma triglycerides (mmol/L), median (Q1-Q3)	0.7 (0.6-0.9)	0.7 (0.6-0.9)	0.8 (0.6-0.9)	.550
Plasma LDL (mmol/L), median (Q1-Q3)	2.1 (1.7-2.4)	2.2 (1.7-2.4)	2.1 (1.8-2.5)	.800
Plasma HDL (mmol/L), median (Q1-Q3)	1.5 (1.3-1.7)	1.4 (1.3-1.6)	1.5 (1.4-1.7)	.853
Plasma non-HDL (mmol/L), median (Q1-Q3)	2.5 (1.9-3.0)	2.5 (1.9-3.0)	2.5 (2.0-3.3)	.629
Plasma hsCRP (mg/L), median (Q1-Q3)	0.3 (0.2-0.7)	0.4 (0.1-0.9)	0.2 (0.2-0.6)	.608
Plasma parathyroid hormone (pmol/L), median (Q1-Q3)	3.9 (3.2-4.6)	4.3 (3.7-4.8)	3.2 (2.8-4.1)	.029
Serum calcium ion (mmol/L), median (Q1-Q3)	1.2 (1.2-1.3)	1.2 (1.2-1.2)	1.3 (1.2-1.3)	.061

All values were based on all 28 (100%) of participants. Body mass index was calculated as weight (kg) divided by the square of height (meters). Differences between groups were tested using a Chi-squared test for categorical variables and a 2-sided Wilcoxon rank-sum test for continuous variables. Abbreviations: AUDIT, alcohol use disorders identification test; HbA_1c_, glycated haemoglobin A_1c_; HDL, high-density lipoprotein; hsCRP, high-sensitivity C-reactive protein; LDL, low-density lipoprotein.

**Fig 2:**
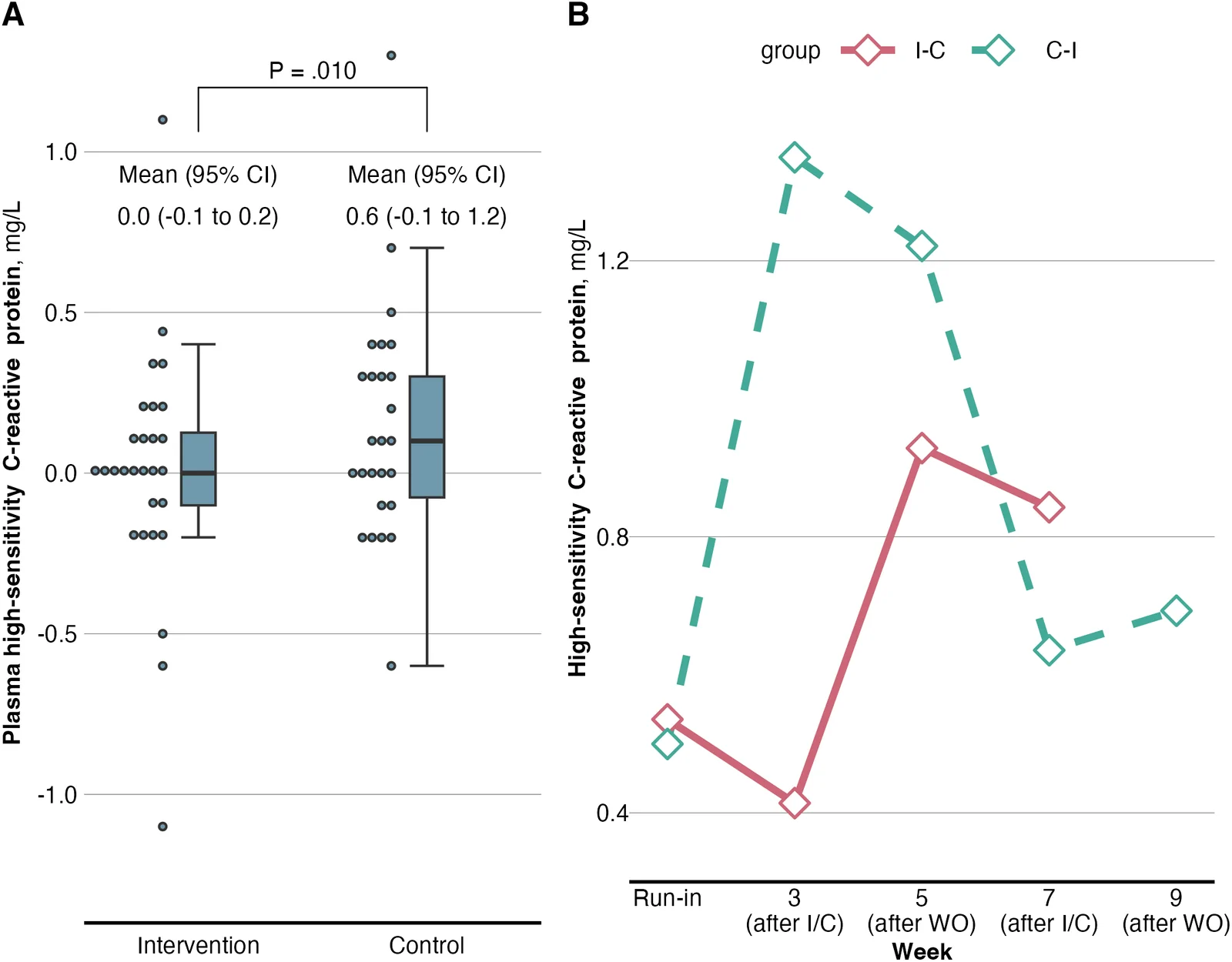
Plasma high-sensitivity C-reactive protein during the intervention and control periods. (A) Box plot of the mean change in high-sensitivity C-reactive protein between the run-in and the end of the intervention and control periods, respectively. (B) Line graph of high-sensitivity C-reactive protein during the intervention then control and control then intervention group during each 2-week period. The difference between the control vs intervention period consisted mainly of higher values during the control period of the control-then-intervention group. For the control period, outliers at 4.4 and 8.0 mg/L are not visualized. Abbreviations: C-I, control then intervention; I-C, intervention then control; I/C, intervention/control; WO, wash-out. No missing values were recorded for any measurement, except for 1 (3.6%) missing plasma insulin value during the intervention period.

Comparing plasma hsCRP values between the intervention and control period, it increase by 32.5% (95% CI −9.7 to 74.6) vs 311.5% (95% CI −40.8 to 663.7), *P* = .045 (Fig 2). However, no significant differences between groups in changes in plasma glucose, blood glycated haemoglobin, serum insulin, plasma lipids, plasma parathyroid hormone and serum calcium ion levels were detected (Table 2). Individual hsCRP trajectories showed substantial variability and no clear sex-specific pattern (S1 Fig).

**Table 2:** Mean percentage difference in blood sample values between the run-in period and the end of the intervention and control periods, respectively, and the difference between those, in 28 healthy adults.

	Mean (95% CI) percentage difference between the run-in period and the end of the intervention period, n = 28	Mean (95% CI) percentage difference between the run-in period and the end of the control period, n = 28	*P*
Plasma glucose	−2.13 (−5.29 to 1.04)	-1.90 (-5.11 to 1.31)	.839
Blood glycated haemoglobin A_1c_	0.16 (−1.42 to 1.74)	1.06 (−0.51 to 2.64)	.256
Serum insulin	15.8 (−3.5 to 35.1)	15.0 (−7.7 to 37.7)	.915
Plasma total cholesterol	−0.78 (−4.06 to 2.51)	1.81 (−2.14 to 5.77)	.258
Plasma triglycerides	4.7 (−10.8 to 20.2)	1.6 (−11.8 to 15.1)	.692
Plasma LDL	−0.53 (−6.68 to 5.62)	4.48 (−1.38 to 10.34)	.152
Plasma HDL	−0.07 (−4.54 to 4.40)	0.21 (−5.23 to 5.66)	.903
Plasma non-HDL	−0.52 (−5.22 to 4.18)	3.45 (−1.68 to 8.57)	.185
Plasma hsCRP	32.5 (−9.7 to 74.6)	311.5 (−40.8 to 663.7)	.045
Plasma parathyroid hormone	6.50 (−9.83 to 22.84)	7.57 (−6.49 to 21.63)	.646
Serum calcium ion	−1.03 (−1.58 to −0.49)	−0.62 (−1.41 to 0.16)	.096

All samples were drawn after fasting. Results for blood samples are presented as the mean (95% CI) change between the run-in period and the end of the intervention and control periods, respectively. Difference between the intervention and control period was tested using a 2-sided paired *t* test for variables with a normal distribution and a paired Wilcoxon signed-rank test for variables with a skewed distribution (blood glycated haemoglobin, serum insulin, plasma triglycerides, plasma hsCRP, and plasma parathyroid hormone). Abbreviations: HDL, high-density lipoprotein; hsCRP, high-sensitivity C-reactive protein; LDL, low-density lipoprotein.

**Fig 3:**
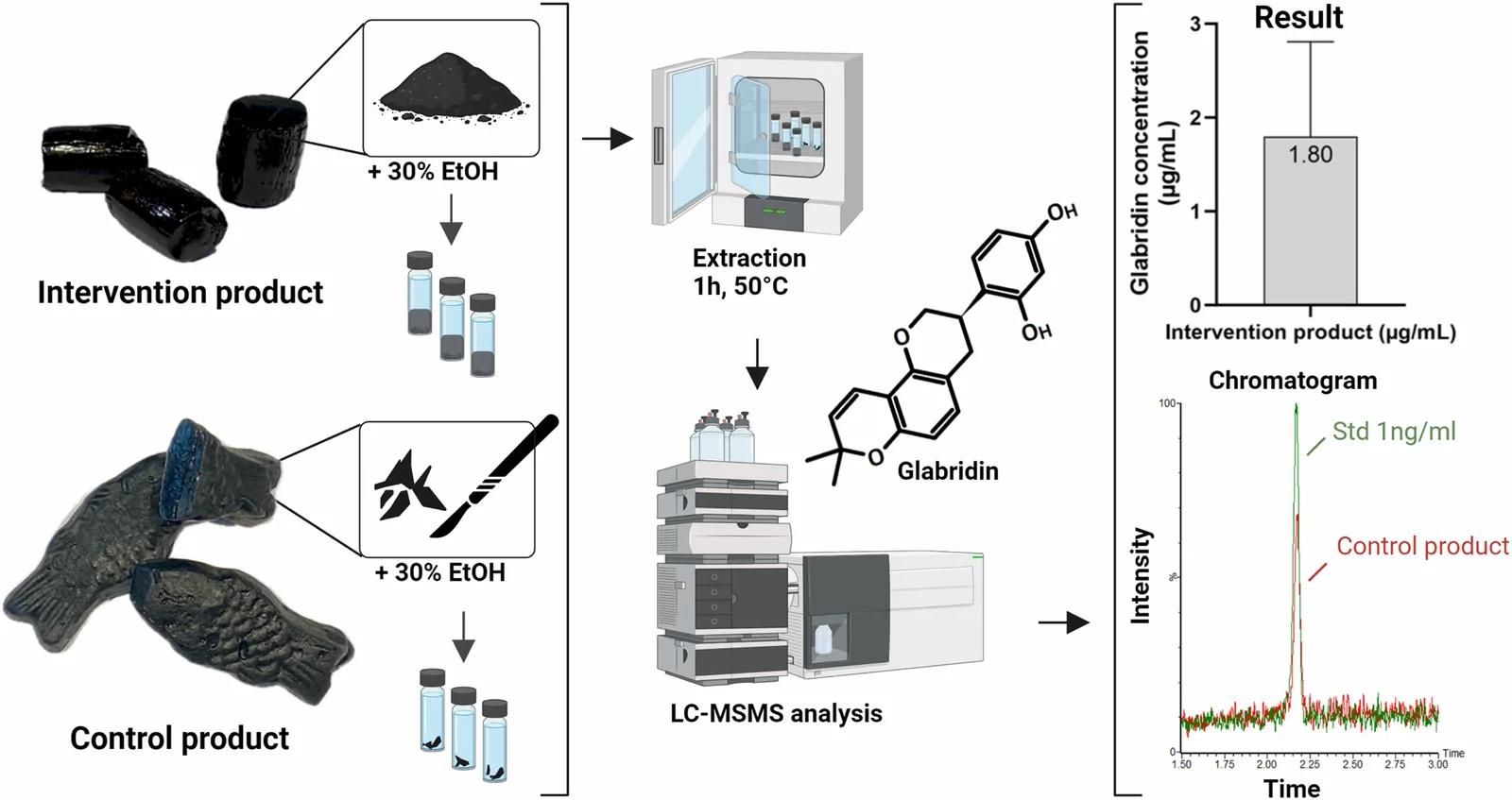
Quantification of glabridin. A confectionary each from the control and intervention products were cut in small pieces or grinded. The confectionary samples (in triplicates) were mixed with 30% ethanol and incubated for 1h at 50°C. The samples were then analysed by LC-MSMS. The quantification result for the intervention product is shown in a bar chart whereas overlayed chromatograms (representative sample of the control product and the lowest calibrator [1 ng/mL]) are presented to show a qualitative presence of glabridin in the sample chemical structure of glabridin is also visualized. Illustration created with BioRender.com. Abbreviations: EtOH, ethanol; LC-MSMS, liquid chromatography tandem mass spectrometry; Std, calibration standard.

The measured concentration of glabridin for the intervention product was 1.80 ± 1.01 µg/mL, corresponding to 0.25 ± 0.14 µg glabridin per mg liquorice after accounting for the extraction. Thus, the 3.3-gram dose of the intervention product contained 0.82 ± 0.46 mg of glabridin. In contrast, only trace levels of glabridin, below the lowest calibrator at 1 ng/ml, were detected but could not be quantified in the control product (Fig 3).

## Discussion

In this secondary analysis of a cross-over study investigating the effects of short-term low-dose liquorice ingestion, an attenuation of an increase in hsCRP was observed, but no change in glycaemic, lipid values, or calcium metabolism was noted.

### The metabolic effects of liquorice in young adults

The study showed that a daily consumption of 3.3 grams of liquorice attenuated an observed increase in plasma hsCRP but did not alter glycaemic levels, lipid levels, or calcium metabolism despite glabridin presence at a concentration of 0.82 mg.

Our study is the first reporting on the effects of whole liquorice intake on glucose or HbA_1c_ levels in vivo in humans. We showed that a daily liquorice intake during a 2-week period did not affect glycaemic or blood lipid levels when compared to the control product. Although plasma hsCRP increased more during the control vs intervention period, it did not change in absolute terms during either period, indicating that this may reflect normal intra-individual variability rather than a true intervention effect.

Further, we did not observe any effect on glycaemic markers. However, this may relate to that the measured marker, HbA_1c,_ reflects the average plasma glucose of the past 8–12 weeks [27], 4–6 times longer than the intervention period. Thus, any future study of glycaemic markers should be of a duration of at least 8 weeks and/or use other measurements, e.g., continuous glucose monitoring devices, to more precisely evaluate the effects on glycemia.

We found no effect on plasma lipid levels, including total cholesterol and LDL. This is in line with a previous study using liquorice flavonoid oil with a glabridin content up to 14 times higher than in our study [22]. However, a study using capsules with *Glycyrrhiza glabra* extract (free from glycyrrhizic acid), with a glabridin content more than 70 times higher than in our study, found that plasma cholesterol and LDL decreased after 6 months. However, this was in comparison to a control product which itself contained more than 6 times the glabridin used in our study [20,21]. Thus, although glabridin by itself may be beneficial for blood lipids, it is unlikely that whole liquorice can be used for such benefits without concomitant unsafe effects on BP.

In our study, hsCRP numerically increased during both periods, but the increase was attenuated during the intervention vs control period. The effect of whole liquorice on hsCRP have not previously been studied in humans. In mice, studies have shown antioxidant and anti-inflammatory effects of liquorice, including inhibition of endothelial cell adhesion molecule expression after tumour necrosis factor-alpha stimulation [6,16,17]. Ex vivo, 1 study showed that LDL from humans was more resistant to oxidation after vs before 2 weeks of liquorice ingestion [8]. Thus, the effect seen in our study could relate to an anti-inflammatory effect of glabridin during the intervention period, followed by a relative increase in hsCRP during the ensuing control period. However, since hsCRP increased numerically in both periods, and individual trajectories showed substantial variability with no clear sex-specific pattern, a random effect cannot be ruled out. Furthermore, we cannot determine if this effect was due to glabridin, or other constituents of liquorice.

We found no effect on plasma PTH or serum calcium ion levels. This is contrasting to a previous study, where a daily intake of 3.5 grams of pure liquorice for 56 days increased plasma PTH in 9 healthy women in Italy. There, serum calcium levels were reported unchanged but albumin-corrected or ionized calcium was not assessed [19]. Given the null effect in our study, this indicates that the effect on plasma PTH may occur beyond 2 weeks.

### Quantification of Glabridin

We present a validated LC-MSMS method for quantification of glabridin that may be used for quantifying the concentration of glabridin in confectionaries. However, sampling from confectionaries proved to be difficult due to their varying consistencies and their possible inhomogeneous nature which may also influence the extraction rate. The glabridin concentration may likely also vary from batch to batch. Further testing might be needed to account for these variations.

### Strengths, limitations, and recommendation for future studies

Our study has some strengths. The design was randomized and controlled, and the dose of whole liquorice intake corresponded to the current upper limit of recommended intake. As intervention, we used whole liquorice, which is the product consumed by most people. Although the control product did not allow blinding, we used a confectionary matched as closely as possible in taste and colour to the whole liquorice. However, it also has some limitations. The sample size and time frame were designed to answer the original study’s primary aim and may have been underpowered for the current analyses. Although the crossover design reduces the impact of between-group differences by allowing each participant to serve as their own control, some baseline imbalances were present between the randomized sequences and their influence on the results cannot be completely excluded. In addition, the apparent difference in hsCRP between intervention and control periods was driven mainly by one randomized sequence, raising the possibility of a sequence effect, period effect, or random variation. Therefore, the finding should be interpreted cautiously and requires confirmation in larger studies. Due to the heterogenous nature of liquorice confectionaries and their multiple components, we cannot determine whether the effects on inflammation are due to glabridin, or if other components of liquorice, including glycyrrhizic acid and its effects on the renin-angiotensin-aldosterone system, may have influenced the results. Even though glabridin has previously been suggested as an anti-inflammatory compound [6–8] and was proven present in the intervention product, the effect observed in our study may have been related to intra-individual variability rather than a true intervention effect. The amount of liquorice consumed by the participants was for ethical purposes equal to the currently recommended safe upper level of intake of glycyrrhizic acid [3], but glabridin may be less potent and require higher doses than those administered to determine its effects. Thus, although we can determine that a daily intake of 3.3 grams of liquorice does not benefit metabolic markers after 2 weeks, our study cannot rule out an effect of isolated glabridin at higher doses or after longer duration. However, knowing that even this low amount of liquorice increases BP, it would not be ethical to use higher doses or extend the study duration using whole liquorice [3]. We suggest that future studies on the effect of glabridin, or any other potentially bioactive substances present in liquorice, is performed using products free from glycyrrhizic acid.

## Conclusion

In conclusion, intake of whole liquorice confectionaries at a dose known to elevate BP was not associated with changes in glycaemic or lipid markers, but potentially health-beneficial effects on inflammation were observed. Further studies are warranted to better characterize this effect. While our study does not rule out potential metabolic benefits at higher doses or longer exposures, it suggests that whole liquorice confectionaries are unlikely to provide such benefits without concomitant detrimental cardiovascular effects due to BP increases.

## Supporting information

S1 Table Previous clinical trials on liquorice and glabridin. (DOCX)

S2 Table Product specifications from producer. (DOCX)

S1 Fig Individual high-sensitivity C-reactive protein (hsCRP) values before and after the intervention and control periods, stratified by sex. (TIF)

## Abbreviations

BP: blood pressure
HbA_1c_: glycated haemoglobin A_1c_
hsCRP: high-sensitivity C-reactive protein
LDL: low-density lipoprotein
LC-MSMS: liquid chromatography tandem mass spectrometry
PTH: parathyroid hormone.
