# Supplementary material for: Glabridin content and metabolic effects of liquorice confectionaries: A secondary analysis of a randomized crossover trial in young adults
Source: PLoS One. 2026 Sep 22;21(9):e0358710. doi: 10.1371/journal.pone.0358710 (PMC13596820; doi:10.1371/journal.pone.0358710)
Supplement: Supplementary file 1 [file pone.0358710.s001.docx]

Glabridin content and metabolic effects of liquorice confectionaries: a secondary analysis of a randomized crossover trial in young adults

**S1 Table. Previous clinical trials on liquorice and glabridin.**

| Title | Year | Subjects | Design | Results |
| --- | --- | --- | --- | --- |
| Clinical safety of licorice flavonoid oil (LFO) and pharmacokinetics of glabridin in healthy humans[22] | 2007 | N = 117 | Randomized, placebo-controlled, single-blind study of 300 vs 600 vs 1200 mg liquorice flavonoid oil (containing 1% glabridin e.g., 3, 6 and 12 mg, respectively) daily vs placebo for 1 and 4 weeks. | No change in blood cell count, albumin, liver enzymes, creatinine, lipid levels, glucose, sodium, potassium, or chloride. |
| The effect of an endogenous antioxidant glabridin on oxidized LDL[20] | 2008 | N = 22 | Random-order, double-blinded cross-over. Subjects ingested capsules with either a *Glycyrrhiza glabra* extract containing 60 mg glabridin or a placebo product containing 5 mg glabridin. | After 6 months, plasma cholesterol and oxidation of LDL was reduced. |
| Antioxidant effect of polyphenolic glabridin on LDL oxidation[21] | 2009 | N = 19 or 22 (19 in methods, 22 in results) |  | After 6 months, plasma cholesterol and oxidation of LDL was reduced. |
