# Supplementary material for: Glabridin content and metabolic effects of liquorice confectionaries: A secondary analysis of a randomized crossover trial in young adults
Source: PLoS One. 2026 Sep 22;21(9):e0358710. doi: 10.1371/journal.pone.0358710 (PMC13596820; doi:10.1371/journal.pone.0358710)
Supplement: Supplementary file 2 [file pone.0358710.s002.docx]

Glabridin content and metabolic effects of liquorice confectionaries: a secondary analysis of a randomized crossover trial in young adults

**S2 Table. Product specifications from producer.**

|  | Intervention product | Control product |
| --- | --- | --- |
| Ingredients | Liquorice extract | Maltitol, starch (corn), E1420 (thickener), ammonium chloride, water, flavourings, E153 (colour) |
| Nutritional values per 100 grams |  |  |
| Energy, kcal | 320 | 369 |
| Fat (of which saturated), g | 0.4 (0.05) | 0.9 (0.8) |
| Carbohydrates (of which sugars), g | 64 (4) | 80.5 (0) |
| Protein, g | 1 | 1.2 |
| Salt, g | 0.03 | 0.05 |
